# The Editor's Letter-Box

**Published:** 1912-07-06

**Authors:** John Anderson

**Affiliations:** Leonard Willox Esq., M.B., Ch.B., General Hospital, Nottingham.


National Health Insurance Commission,

SIR,—In reply to your letter of the 19th instant I am directed by the National Health Insurance Commission (Eng.) to state that the Commissioners are advised that the term "inmate" does not include the staff of your hospital, but only patients. The resident medical officers would, therefore, appear to be liable to compulsory insurance under Part I. of the National Insurance Act where the rate of remuneration does not exceed £160 a year.

In the calculation of remuneration for the purposes of the Act the value of board and lodging, if provided, will be taken into account, but no account is to be taken of private income, although if such private income not dependent on the personal exertions of the recipient amounted to £26 a year or more it would enable him, should he so desire, to obtain a certificate exempting him from the liability to become insured.

The granting of such a certificate would not, however, relieve his employer from the liability to pay the employer's share of the contribution. The rates of contribution are set out in Part VI. of the enclosed memorandum.

Graduates who as students are attending post-graduate courses at colleges and universities are not employed within the meaning of the Act, and are therefore outside the compulsory provisions of the Act. A set of official explanatory leaflets is enclosed.

I am, Sir, your obedient servant,
(Signed) JOHN ANDERSON.

Leonard Willox, Esq., M.B., Ch.B.,
General Hospital, Nottingham.

